# Heterozygous knockout of *Bile salt export pump* ameliorates liver steatosis in mice fed a high-fat diet

**DOI:** 10.1371/journal.pone.0234750

**Published:** 2020-08-12

**Authors:** Kazuya Okushin, Takeya Tsutsumi, Kazuhiko Ikeuchi, Akira Kado, Kenichiro Enooku, Hidetaka Fujinaga, Naoko Yamauchi, Tetsuo Ushiku, Kyoji Moriya, Hiroshi Yotsuyanagi, Kazuhiko Koike

**Affiliations:** 1 Department of Gastroenterology, Graduate School of Medicine, The University of Tokyo, Tokyo, Japan; 2 Department of Infection Control and Prevention, Graduate School of Medicine, The University of Tokyo, Tokyo, Japan; 3 Division of Infectious Diseases, Advanced Clinical Research Center, The Institute of Medical Science, The University of Tokyo, Tokyo, Japan; 4 Department of Pathology, Graduate School of Medicine, The University of Tokyo, Tokyo, Japan; Nihon University School of Medicine, JAPAN

## Abstract

The incidence of nonalcoholic steatohepatitis (NASH) is increasing worldwide, including in Asian countries. We reported that the hepatic expression of bile salt export pump (BSEP) was downregulated in patients with NASH, suggesting that BSEP is involved in the pathogenesis of NASH. To identify the underlying mechanism, we analyzed *Bsep* heterozygous knock-out (*Bsep*^*+/-*^ mice) and wild-type (WT) C57BL/6J mice fed a high-fat diet (HFD) (32.0% animal fat) or normal diet. We examined histological changes, levels of hepatic lipids and hepatic bile acids, and expression of genes related to bile acid and cholesterol metabolism. HFD-fed *Bsep*^*+/-*^ mice exhibited milder hepatic steatosis and less weight gain, compared to HFD-fed WT mice. The concentrations of total bile acid, triglycerides, and cholesterols were reduced in the liver of HFD-fed *Bsep*^+/-^ mice. Regarding hepatic bile acid metabolism, the expression levels of *Farnesoid X receptor* (*Fxr*) and *Multidrug resistance-associated protein 2* were significantly upregulated in HFD-fed *Bsep*^*+/-*^ mice, compared to HFD-fed WT mice. Furthermore, several alterations were observed in upstream cholesterol metabolism in the liver. The expression levels of bile acid metabolism-related genes were also altered in the intestine of HFD-fed *Bsep*^*+/-*^ mice. In conclusion, HFD-fed *Bsep*^*+/-*^ mice exhibited significant alterations of the expression levels of genes related to bile acid and lipid metabolism in both the liver and ileum, resulting in alleviated steatosis and less weight gain. These results suggest the importance of BSEP for maintenance of bile acid and cholesterol metabolism. Further investigations of the involvement of BSEP in the pathogenesis of NASH will provide greater insight and facilitate the development of novel therapeutic modalities.

## Introduction

The incidence of nonalcoholic fatty liver disease (NAFLD) is increasing worldwide, including in Asian countries [1–4]. NAFLD is a spectrum of disease ranging from simple steatosis to nonalcoholic steatohepatitis (NASH). NASH is progressive and is considered a causative factor of cirrhosis, hepatocellular carcinoma, and systemic metabolic disorders [5–7]. However, the pathogenesis of NASH is unclear.

Bile acid is a detergent-like compound that promotes the absorption of dietary lipids. However, bile acid has been recognized as a ligand of nuclear receptor farnesoid X receptor (FXR) [8–10]; it is considered a central factor in systemic metabolic pathways, including glucose and lipid metabolism [11–14]. NAFLD and NASH are also closely related to systemic metabolism. Consequently, bile acid metabolism has received attention as a therapeutic target for NASH [15–19]. Indeed, the FXR ligand obeticholic acid is used clinically [20–22]. In addition, bile acid concentrations are elevated in the hepatocytes and/or plasma of patients with NASH [23–26]. We previously reported that the intrahepatic expression of the bile salt export pump (BSEP) was downregulated during progression of NAFLD, suggesting that BSEP is involved in the pathogenesis of NASH [27]. Here, we investigated the role of BSEP in the pathogenesis of NASH by using mice with a *Bsep* modification.

## Materials and methods

### Animals

*Bsep* homozygous knock-out mice (B6.129S6-Abcb11^tm1Wng^/J) [28] were purchased from The Jackson Laboratory (Bar Harbor, ME, USA). *Bsep* heterozygous knock-out mice (*Bsep*^*+/-*^ mice) were generated from B6.129S6-Abcb11^tm1Wng^/J and wild-type (WT) mice (C57BL/6J) purchased from Charles River Laboratories Japan (Yokohama, Kanagawa, Japan). The mice were housed in a specific pathogen-free environment controlled for temperature and light (25°C, 12-h/12-h light/dark cycle) and observed for signs of distress. The experimental protocols were approved by the Ethics Committee for Animal Experimentation and were conducted in accordance with the Guidelines for the Care and Use of Laboratory Animals of the Department of Medicine, The University of Tokyo (No. P15-078).

WT and *Bsep*^*+/-*^ male mice were both fed a high-fat diet (HFD; HFD32^®^, 32.0% animal fat, CLEA Japan, Inc. Tokyo, Japan) or normal diet (ND; CE-2^®^, CLEA Japan, Inc. Tokyo, Japan) at fixed intervals with fixed amounts beginning at 8 weeks of age and were analyzed at 20 weeks of age. The number of mice of each group are following: ND-fed WT mice (n = 6), HFD-fed WT mice (n = 6), ND-fed *Bsep*^*+/-*^ mice (n = 5), and HFD-fed *Bsep*^*+/-*^ mice (n = 5). All sacrifice procedures were performed by cervical dislocation under anesthesia with isoflurane, and all efforts were made to minimize suffering.

### Quantification of lipids and total bile acids in the liver

Quantification of intrahepatic lipids and total bile acid (TBA) was conducted at Skylight Biotech, Inc. (Akita, Japan). Lipids and TBA were extracted by the Folch method and ethanol, respectively [29]. Triglyceride (TG), total cholesterol (T-CHO), free cholesterol (F-CHO), and TBA concentrations were quantified using the Cholestest^®^ TG, Cholestest^®^ CHO (Sekisui Medical, Tokyo, Japan), Cholescolor Liquid FC (Toyobo, Osaka, Japan), and Total Bile Acids Assay Kit (Diazyme Laboratories, Inc., Poway, CA, USA), respectively. The TG, T-CHO, F-CHO, and TBA levels are presented as mg/g, and were calculated by dividing the amount of detected analyte by the weight of liver tissue.

### Evaluation of hepatic bile acid fractions

The hepatic levels of the major bile acids cholic acid (CA), glycolic acid (GCA), tauro-cholic acid (TCA), tauro-chenodeoxycholic acid (TCDCA), tauro-deoxycholic acid (TDCA), tauro-lithocholic acid (TLCA), and tauro-ursodeoxycholic acid (TUDCA) were determined by liquid chromatography time-of-flight mass spectroscopy (LC-TOFMS); three representative samples were analyzed per group. Metabolite extraction and metabolome analysis were conducted at Human Metabolome Technologies (Tsuruoka, Yamagata, Japan). Briefly, approximately 50 mg of frozen tissue were immersed in 500 μL of 1% formic acid/acetonitrile containing internal standard solution (H3304-1002, Human Metabolome Technologies) at 0°C. The tissue was homogenized three times at 1,500 rpm for 120 s each using a tissue homogenizer (Micro Smash MS100R; Tomy Digital Biology Co., Ltd.) and the homogenate was centrifuged (2,300 g, 4°C, 5 min). The supernatant was mixed with 500 μL of 1% formic acid/acetonitrile and 167 μL of Milli-Q water, passed through a 3-kDa cutoff filter (Nanocep 3K Omega; Pall Corp.) to remove macromolecules, and filtered using a hybrid SPE phospholipid cartridge (55261-U; Supelco) to remove phospholipids. The filtrate was desiccated and resuspended in 100 μL of isopropanol/Milli-Q water for LC-TOFMS analysis using an Agilent LC System equipped with an ODS column (Agilent Technologies). The system was controlled using Agilent G2201AA ChemStation software version B.03.01 (Agilent Technologies) [30]. Peaks were extracted using MasterHands automatic integration software (Keio University, Tsuruoka, Yamagata, Japan) to obtain the *m/z*, peak area, and migration time [31]. Signal peaks corresponding to isotopomers, adducts, and other product ions were compared with a database of known metabolites based on their *m/z* values and migration and retention times. The areas of the annotated peaks were normalized to those of the internal standards to determine the levels of metabolites.

### Quantitative polymerase chain reaction

Total liver RNA was extracted using the ReliaPrep RNA Tissue Miniprep System (Promega, Madison, WI, USA). The levels of messenger RNAs (mRNAs) encoding *Bsep*, *multidrug resistance-associated protein 2* (*Mrp2*), *Na*^*+/*^*taurocholate cotransporter* (*Ntcp*), *Fxr*, *cholesterol 7 alpha-hydroxylase* (*Cyp7a1*), *3-hydroxy-3-methylglutaryl-Coenzyme A reductase* (*Hmgcr*), *low-density lipoprotein receptor* (*Ldlr*), *ATP-binding cassette sub-family G member 5* (*Abcg5*), *sterol regulatory element binding transcription factor 2 (Srebf2)*, *collagen type I*, *alpha 1* (*Col1a1*), *collagen type I*, *alpha 2* (*Col1a2)*, *tissue inhibitor of metalloproteinase 1 (Timp1)*, *matrix metallopeptidase 3 (Mmp3)*, *transforming growth factor*, *beta 1 (Tgfb1)*, *intestinal bile acid-binding protein* (*I-babp*), *transmembrane G protein-coupled receptor 5 (Tgr5)*, *Niemann–Pick C1 like 1* (*Npc1l1*), and the housekeeping gene *glyceraldehyde-3-phosphate dehydrogenase* (*Gapdh*) in liver tissue were determined by fluorescence-based reverse transcriptase-polymerase chain reaction (RT-PCR) using a StepOnePlus™ Real-Time PCR System (Applied Biosystems, Foster City, CA, USA) using the TaqMan Universal PCR Master Mix reagent (Applied Biosystems). The primers and probes (Applied Biosystems) are listed in S1 Table. The mRNA levels were normalized to the level of the endogenous control, *Gapdh*, using a primer and probe for GAPDH (4326317E, Applied Biosystems).

### Histological analyses

All liver samples were examined by a single experienced pathologist (N.Y.) blinded to all background data and study design. Immunohistochemistry was used to evaluate BSEP expression, at the protein level, using the rabbit polyclonal antibody against BSEP (HPA019035, Sigma-aldrich Inc., St. Louis, MO, USA).

### Blood examinations

Blood samples from each mouse were collected from the inferior vena cava. After collection of the whole blood, it was left undisturbed for clotting at room temperature for 30 minutes. Serum was separated by centrifugation at 1,000 x g in a refrigerated centrifuge for 30 minutes. All blood examinations including aspartate aminotransferase (AST), alanine aminotransferase (ALT), alkaline phosphatase (ALP), T-CHO, TG, and glucose were performed at the Oriental Kobo Life Science Laboratory (Nagahama, Japan).

### Statistics

Data are means and standard deviations of the mean unless stated otherwise. Data processing and analysis were performed using JMP Pro 14 software (SAS Institute Japan, Tokyo, Japan). Differences in gene expression levels were evaluated by Student’s t-test. A *p-*value < 0.05 was considered indicative of statistical significance.

## Results

### *Bsep* heterozygous knock-out mice fed a high-fat diet exhibit milder steatosis

In this study, we adopted *Bsep* homozygous knock-out mice (B6.129S6-Abcb11^tm1Wng^/J) [28] which was established as a model of intrahepatic cholestasis. In human, mutations in *BSEP* induce severe intrahepatic cholestasis, known as progressive familial intrahepatic cholestasis type 2 [32, 33]. However, B6.129S6-Abcb11^tm1Wng^/J exhibit milder intrahepatic cholestasis because of the compensatory actions of other P-glycoprotein genes [34]. Because patients with NASH have reduced but not absent expression of BSEP [27], we used *Bsep*^*+/-*^ mice fed an HFD to evaluate the role of BSEP in the pathogenesis of NASH.

**Fig 1:**
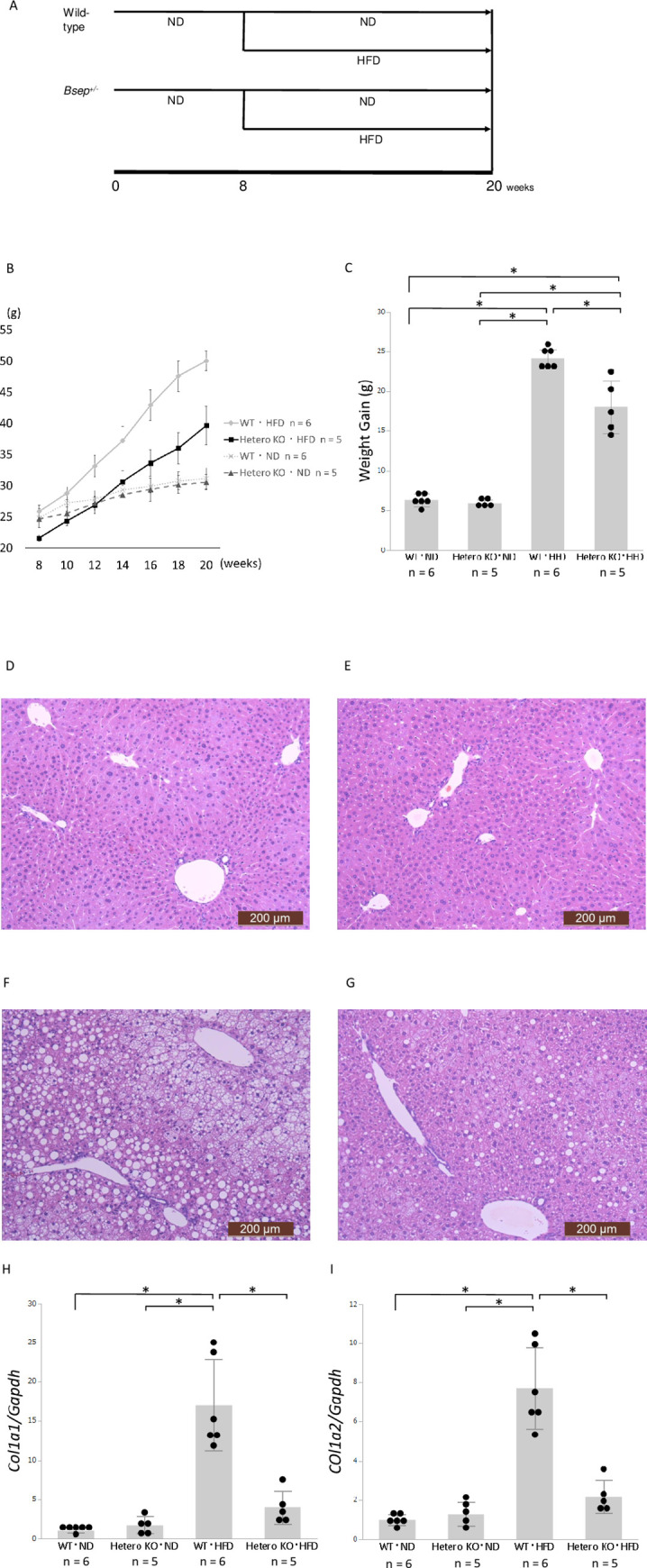
*Bsep*^*+/-*^ and Wild-Type (WT) mice fed with a Normal Diet (ND) or High-Fat Diet (HFD). **A**: Time course. WT mice fed an ND (n = 6) or HFD (n = 6). *Bsep*^*+/-*^ mice fed an ND (n = 5) or HFD (n = 5). **B and C**: Transition of body weight **(B)** and weight gain **(C)**. Values are expressed as means ± standard deviations (n  =  5 or 6). * *p* < 0.05. **D, E, F, and G**: Hematoxylin & eosin staining of representative liver specimens. **D**: ND-fed WT mice. **E**: ND-fed *Bsep*^*+/-*^ mice. **F**: HFD-fed WT mice. **G**: HFD-fed *Bsep*^*+/-*^ mice. Bars: 200 μm (D, E, F, and G). **H and I**: Hepatic expression levels of *Col1a1* and *Col1a2*. Values are expressed as means ± standard deviations (n  =  5 or 6). * *p* < 0.05.

*Bsep*^*+/-*^and WT male mice were fed an HFD or ND beginning at 8 weeks of age, bred for 12 weeks, and sacrificed at 20 weeks of age (n = 5 or 6 mice per group) (Fig 1A). As expected, mice fed an HFD exhibited increased weight gain, compared to those fed an ND. Notably, HFD-fed *Bsep*^*+/-*^ mice exhibited less weight gain, compared to HFD-fed WT mice (18.0 ± 3.3 g *vs*. 24.2 ± 1.13 g, *p* < 0.05, Fig 1B and 1C). In contrast, ND-fed *Bsep*^*+/-*^ and ND-fed WT mice exhibited similar weight gain (6.3 ± 0.8 g *vs*. 5.9 ± 0.4 g, *p* = 0.7112, Fig 1B and 1C).

At 20-week-old, ND-fed WT and *Bsep*^*+/-*^ mice exhibited almost normal histology without steatosis (Fig 1D and 1E). In contrast, HFD-fed mice exhibited steatosis; this was milder in HFD-fed *Bsep*^*+/-*^ mice than in HFD-fed WT mice (Fig 1F and 1G). Regarding inflammation in the liver, interestingly, both ND- and HFD-fed *Bsep*^*+/-*^ mice presented several focal inflammatory lesions (S2A–S2D Fig). HFD-fed WT mice presented slight inflammation mainly in the portal area (Fig 1F) with significant elevation of serum AST and ALT (S1A and S1B Fig). Although significant fibrosis was observed by neither Hematoxylin & eosin (Fig 1D–1G) nor Azan staining (S2E–S2H Fig), HFD-fed WT mice showed higher expression levels of *Col1a1*, *Col1a2*, *Timp1*, and *Mmp3* which are related to hepatic fibrogenesis; in contrast, the expression levels of these genes were markedly suppressed in HFD-fed *Bsep*^*+/-*^ mice (Fig 1H and 1I; S3A and S3B Fig).

### Alterations of bile acids and lipids in the liver

**Fig 2:**
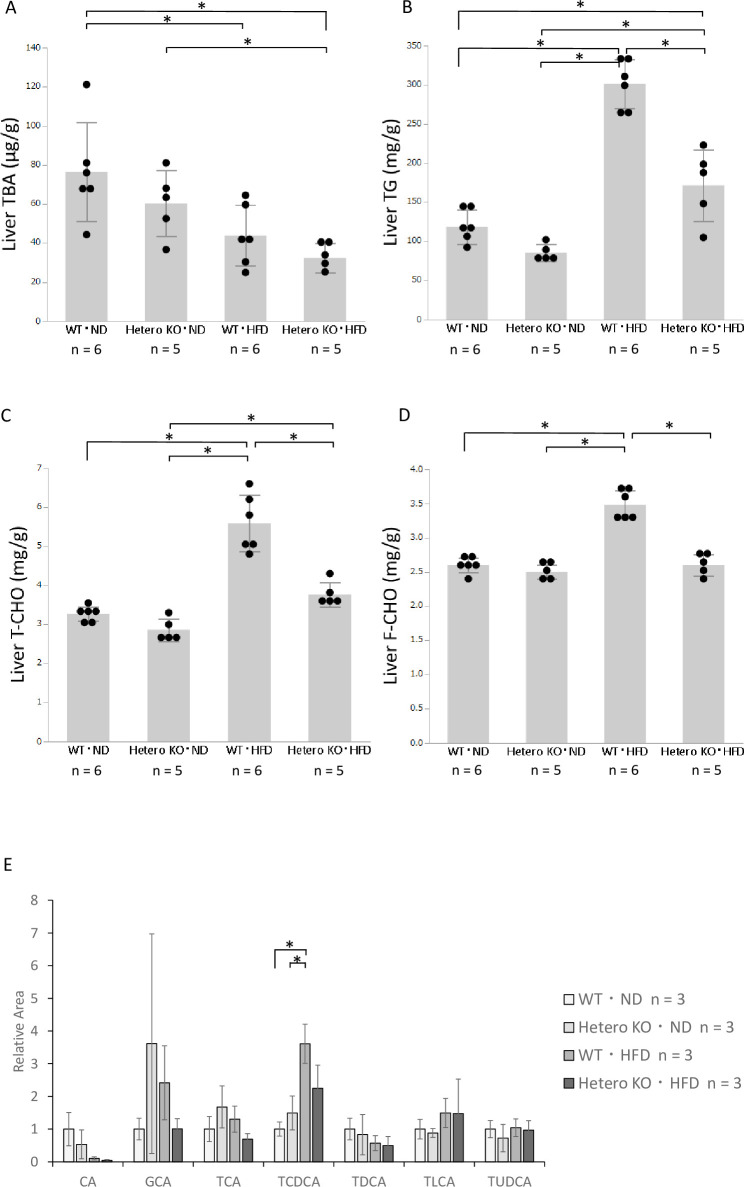
Hepatic concentrations of total bile acids and lipids. **A**: Total bile acid level. B: Triglyceride level. **C and D**: Total and free cholesterol levels. Values are expressed as means ± standard deviations (n  =  5 or 6). **E**: Bile acid fractions in the liver. Values are expressed as means ± standard deviations (n  = 3). * *p* < 0.05.

We quantified the hepatic concentrations of TBA and lipids. First, based on data from human liver biopsy samples [27], we hypothesized that *Bsep*^+/-^ mice would have excess bile acid in the liver. However, the total bile acid levels in the liver were reduced in *Bsep*^+/-^ mice, compared with WT mice, in both diet groups (Fig 2A). Next, we analyzed the hepatic bile acid fractions by LC-TOFMS. The results were not statistically significant for each fraction because of the small sample size (n = 3 per each group). Therefore, we focused on the levels of primary bile acids (CA, GCA, TCA, and TCDCA) and secondary bile acids (TDCA, TLCA, and TUDCA). The levels of primary bile acids showed a decreasing tendency in HFD-fed *Bsep*^+/-^ mice, compared with HFD-fed WT mice; however, the levels of secondary bile acids did not differ in these mice (Fig 2E).

The hepatic levels of TG, T-CHO, and F-CHO were increased in the HFD-fed mice, compared with ND-fed WT mice. However, all levels were significantly reduced in HFD-fed *Bsep*^+/-^ mice. Triglyceride levels in the liver were reduced in HFD-fed *Bsep*^*+/-*^ mice, compared with HFD-fed WT mice (171.6 ± 45.7 mg/g *vs*. 301.0 ± 31.1 mg/g, *p* < 0.05, Fig 2B). The hepatic T-CHO and F-CHO levels were reduced in HFD-fed *Bsep*^*+/-*^ mice, compared with HFD-fed WT mice (3.76 ± 0.30 mg/g *vs*. 5.58 ± 0.73 mg/g and 2.6 ± 0.16 mg/g *vs*. 3.48 ± 0.20 mg/g, respectively, both *p* < 0.05, Fig 2C and 2D). These results were consistent with the histologically ameliorated hepatic steatosis in HFD-fed *Bsep*^*+/-*^ mice. The results of blood examinations including T-CHO, TG, and glucose were presented in S1 Fig. Serum T-CHO and glucose were elevated in HFD-fed mice compared with ND-fed mice (S1D and S1F Fig). Serum TG was decreased in HFD-fed WT mice compared with other groups (S1E Fig).

### Alterations of bile acid metabolism in *Bsep* heterozygous knock-out mice fed an HFD

Bile acid metabolism is regulated by a feedback system comprising a synthetic pathway and several membrane transporters. FXR is a key player in bile acid metabolism, which is activated by bile acid. CYP7A1 is the rate-limiting enzyme in bile acid synthesis. Bile acid is transported through the portal vein, hepatocytes, and bile canaliculi by several membrane transporters. In the liver, BSEP with support from MRP2 exports bile acid from hepatocytes to bile canaliculi; NTCP is principally responsible for the uptake of bile acid from the portal vein into hepatocytes. We examined alterations in the expression levels of genes related to bile acid metabolism.

**Fig 3:**
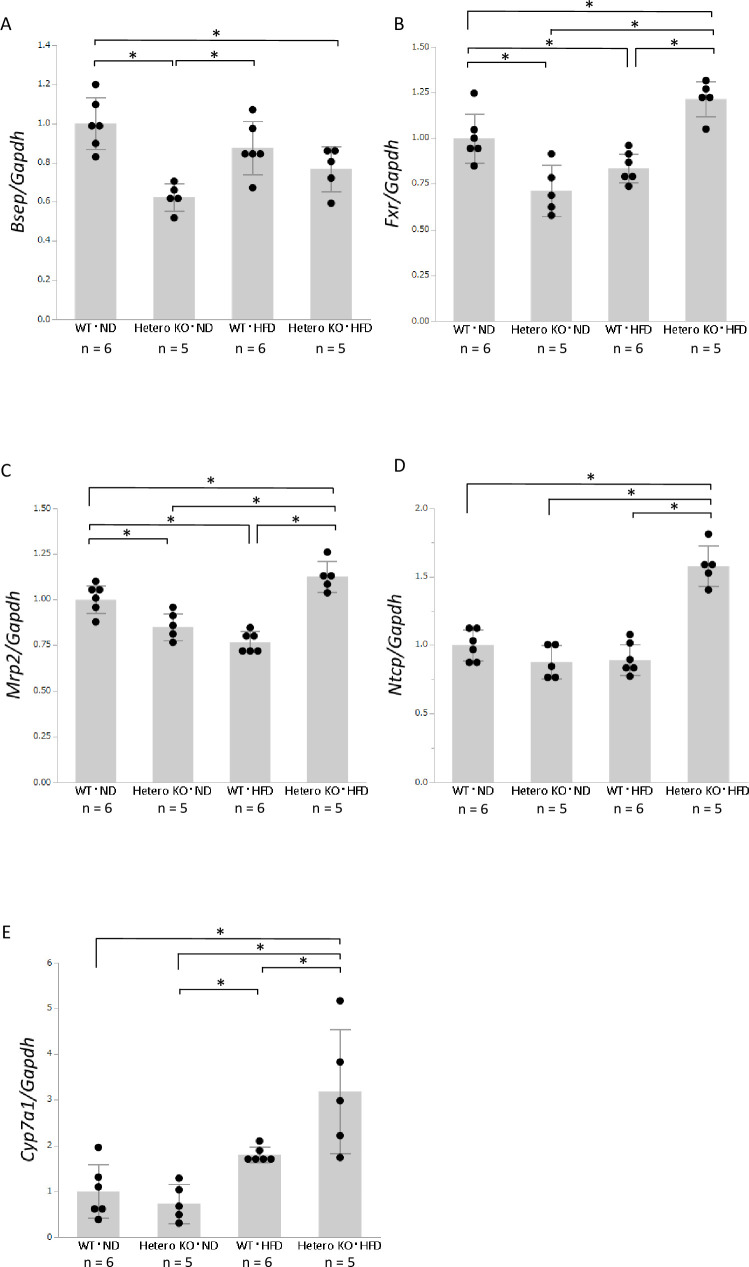
Hepatic expression levels of genes related to bile acid metabolism, as determined by real-time PCR. **A**: mRNA expression levels of *Bsep*. **B**: mRNA expression levels of *Fxr*. **C**: mRNA expression levels of *Mrp2*. **D**: mRNA expression levels of *Ntcp*. **E**: mRNA expression levels of *Cyp7a1*. Values are expressed as means ± standard deviations (n  =  5 or 6). * *p* < 0.05.

We first confirmed the reduction of *Bsep* expression in *Bsep*^+/-^ mice (Fig 3A). The *Bsep* mRNA level was significantly downregulated in ND-fed *Bsep*^+/-^ mice, compared with ND-fed WT mice. In addition, HFD-fed *Bsep*^+/-^ mice exhibited a slight reduction in *Bsep* mRNA level, compared with HFD-fed WT mice. We also evaluated BSEP expression at the protein level by immunohistochemistry. BSEP is mainly expressed at cellular membrane of each hepatocyte, and BSEP expression in ND- or HFD-fed *Bsep*^+/-^ mice (S4A and S4C Fig) was weaker than that in WT mice (S4B and S4D Fig). We next focused on FXR, a main regulator of bile acid metabolism. Although hepatic expression of *Fxr* was lower in ND-fed *Bsep*^+/-^ mice than in ND-fed WT mice, it was higher in HFD-fed *Bsep*^+/-^ mice than in HFD-fed WT mice (Fig 3B). Expression levels of the export transporter *Mrp2*, which is positively regulated by FXR, were similar to those of *Fxr* (Fig 3C). *Ntcp*, a bile acid uptake transporter, was upregulated only in HFD-fed *Bsep*^+/-^ mice (Fig 3D).

Finally, we examined expression levels of the rate-limiting enzyme of bile acid synthesis, *Cyp7a1*. The expression levels of *Cyp7a1* were similar in ND-fed *Bsep*^+/-^ and WT mice; they were increased by the HFD, but were higher in HFD-fed *Bsep*^*+/-*^ mice than in HFD-fed WT mice (Fig 3E).

### Alterations of cholesterol metabolism in *Bsep* heterozygous knockout mice fed an HFD

Cholesterol metabolism is closely linked to bile acid metabolism, because bile acid is synthesized from cholesterol. Therefore, we next examined the expression levels of genes related to cholesterol metabolism.

**Fig 4:**
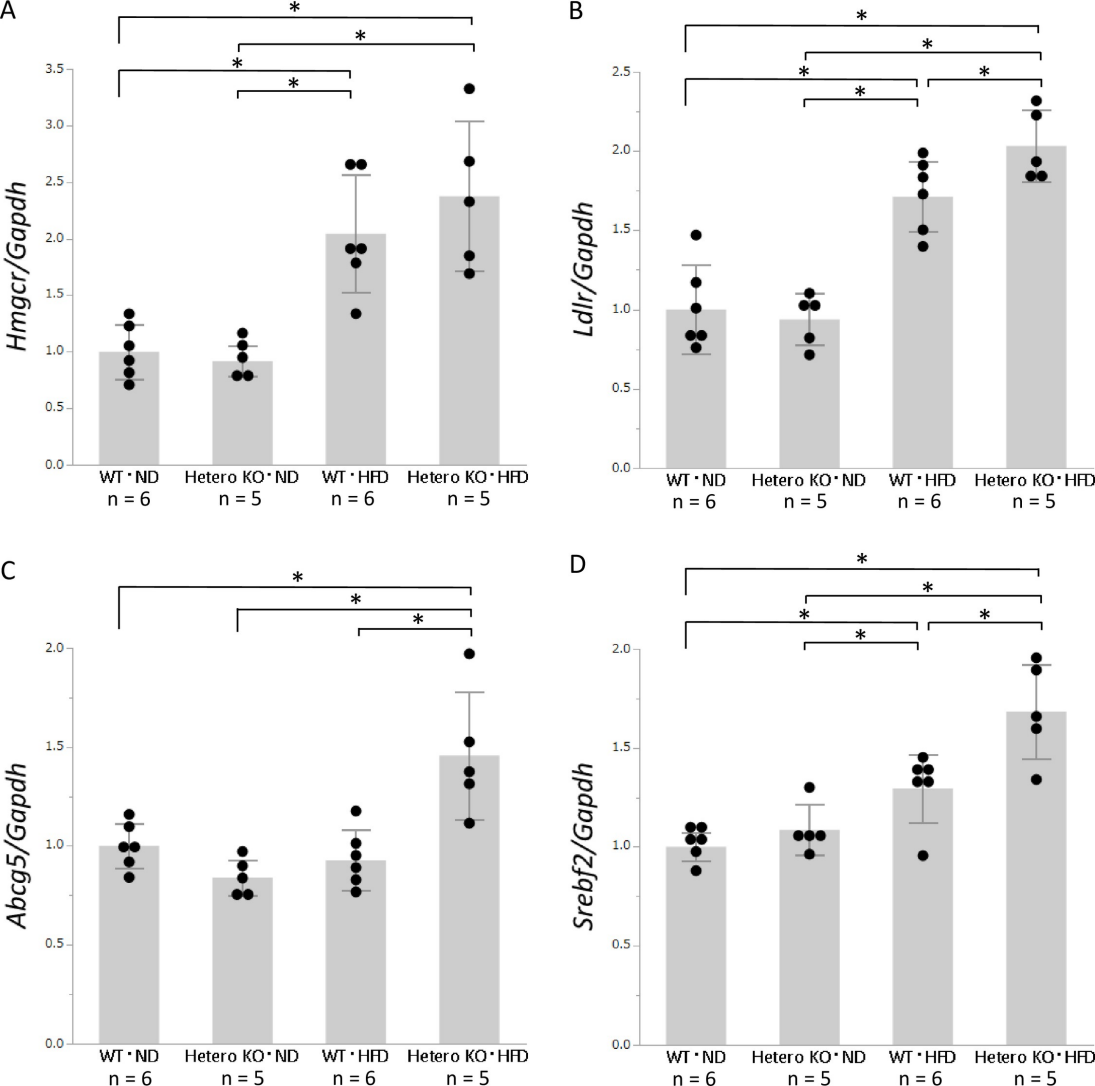
Hepatic mRNA expression levels of genes associated with cholesterol metabolism, as determined by real-time PCR. **A**: mRNA expression levels of *Hmgcr*. **B**: mRNA expression levels of *Ldlr*. **C**: mRNA expression levels of *Abcg5*. **D**: mRNA expression levels of *Srebf2*. Values are expressed as means ± standard deviations (n  =  5 or 6). * *p* < 0.05.

The expression of *Hmgcr*, the rate-limiting enzyme of cholesterol synthesis, was significantly upregulated in the HFD-fed mice, compared with ND-fed mice; however, it was not significantly different between *Bsep*^+/-^ and WT mice (Fig 4A). *Ldlr*, the main cholesterol uptake transporter, was also significantly upregulated in HFD-fed mice, compared with ND-fed mice. In addition, it was significantly upregulated in HFD-fed *Bsep*^+/-^ mice, but not ND-fed *Bsep*^+/-^ mice, compared with the corresponding WT mice (Fig 4B). *Abcg5*, a cholesterol exporter, was similarly expressed among ND-fed *Bsep*^+/-^ mice, ND-fed WT mice, and HFD-fed WT mice, but was significantly upregulated in HFD-fed *Bsep*^+/-^ mice (Fig 4C). *Srebf2*, a regulator of cholesterol metabolism, was upregulated in HFD-fed *Bsep*^+/-^ mice, compared with ND-fed *Bsep*^+/-^ mice (Fig 4D). SREBP2 senses reductions in the hepatic cholesterol level and stimulates HMGCR and LDLR to maintain the cholesterol pool. The abovementioned alterations in gene expression are consistent with compensation for a reduced hepatic cholesterol level.

### Effect on bile acid and cholesterol metabolism in the ileum of *Bsep* heterozygous knockout mice

Alterations of the expression levels of genes related to bile acid and cholesterol metabolism in the liver may partly explain the milder steatosis and lesser weight gain observed in HFD-fed *Bsep*^*+/-*^ mice. Because intrahepatic downregulation of *Bsep* might affect extrahepatic metabolism, we examined its expression in the mouse ileum, where bile acid and lipids are reabsorbed.

**Fig 5:**
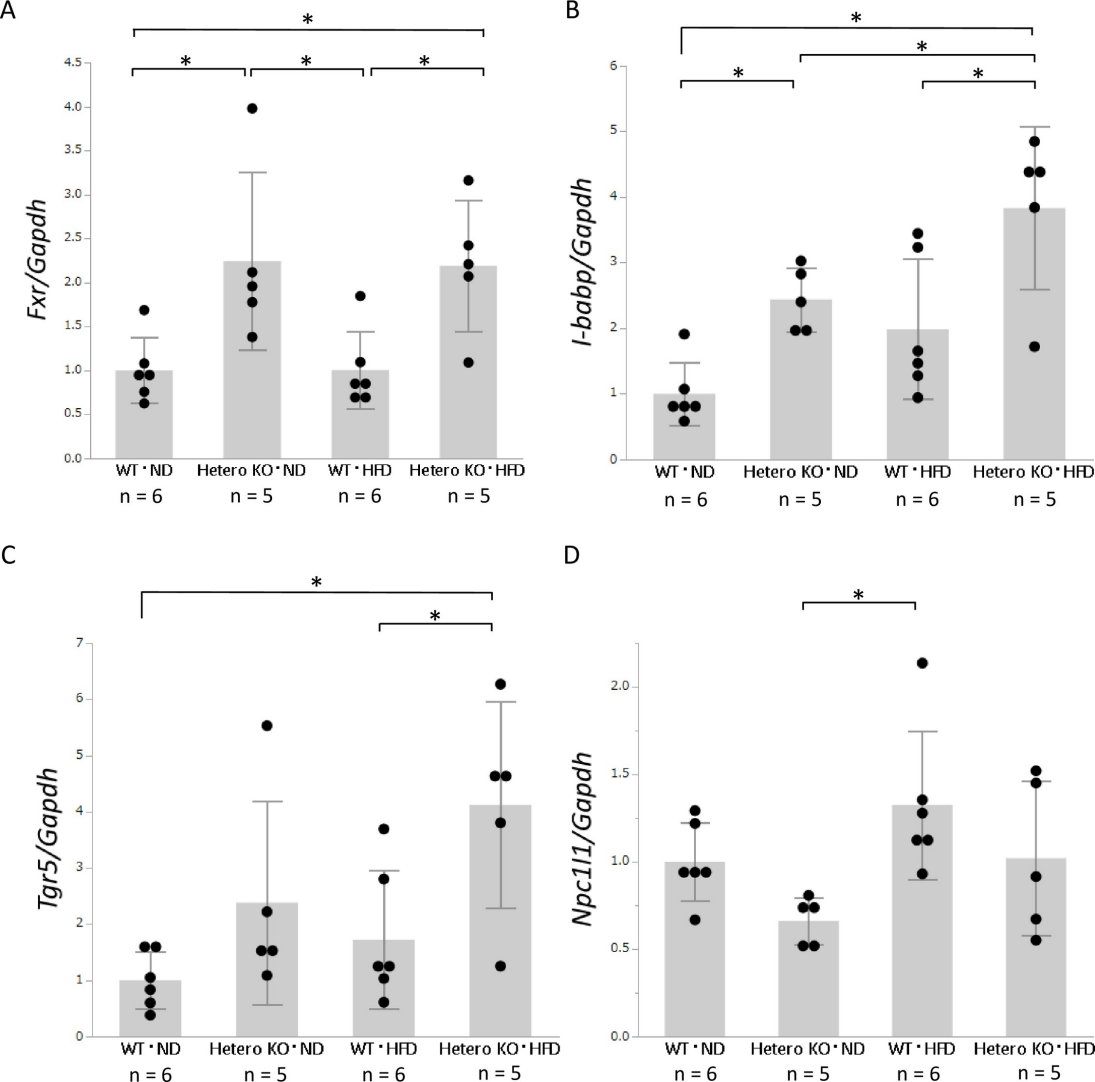
Intestinal mRNA expression levels of genes associated with bile acid and cholesterol metabolism, as determined by real-time PCR. **A**: mRNA expression levels of *Fxr*. **B**: mRNA expression levels of *I-babp*. **C**: mRNA expression levels of *Tgr5*. **D**: mRNA expression levels of *Npc1l1*. Values are expressed as means ± standard deviations (n  =  5 or 6). * *p* < 0.05.

FXR plays a central role in bile acid metabolism in the ileum. Intestinal FXR is activated by bile acid secreted from the liver to the intestinal tract. *Intestinal bile acid-binding protein* (*I-babp*), also known as *fatty acid binding protein 6* (*Fabp6*), is activated by FXR and transports bile acid through intestinal cells. The expression levels of *Fxr* and *I-babp* were significantly upregulated in *Bsep*^*+/-*^ mice, compared with WT ND- and HFD-fed mice (Fig 5A and 5B).

TGR5 is a bile acid receptor that is expressed in several organs, including the intestines; it plays a central role in energy expenditure and glucose metabolism [35]. Notably, *Tgr5* expression was significantly upregulated in ND- and HFD-fed *Bsep*^*+/-*^ mice, compared with WT mice (Fig 5C).

The expression of *Npc1l1*, a cholesterol uptake transporter in the ileum, tended to be lower in HFD-fed *Bsep*^*+/-*^ mice than in HFD-fed WT mice, although this difference was not statistically significant (Fig 5D). This difference may contribute to the alleviation of hepatic steatosis and reduction of the intrahepatic bile acid level observed in HFD-fed *Bsep*^*+/-*^ mice.

## Discussion

**Fig 6:**
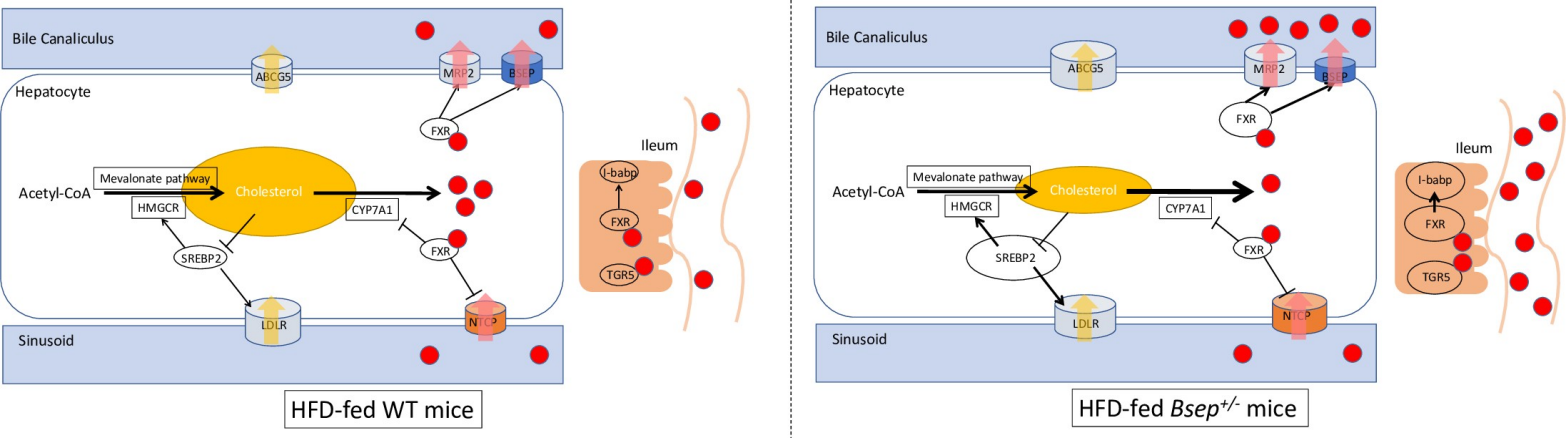
Graphical summary of the metabolic alterations observed in this study. Several alterations in bile acid and cholesterol metabolism are observed in both the liver and ileum of HFD-fed WT mice and *Bsep*^*+/-*^ mice. *Bsep* heterozygous KO induces MRP2 activation via FXR. As a result, export of bile acids through MRP2 is accelerated, finally resulting in reduction of intrahepatic bile acid pool. In the ileum, acceleration of bile acid flow from the liver activate bile acid receptors such as FXR and TGR5. In the liver, CYP7A1 is activated to compensate the reduction of intrahepatic bile acid pool, resulting in conversion of cholesterols to bile acid. **Red circle**: bile acid, **Yellow ellipse**: cholesterol, **Red arrow**: bile acid flow, **Yellow arrow**: cholesterol flow. Sizes of each figure present alterations of gene expressions or amounts of compounds.

In this study, we examined the effect of reduced BSEP expression and found that HFD-fed *Bsep*^*+/-*^ mice exhibited attenuation of hepatic steatosis, together with a reduced level of bile acid. Several alterations in bile acid and cholesterol metabolism were also observed in both the liver and ileum (Fig 6). Some of these manifestations in mice were unexpectedly different from those observed in human patients. These alterations suggest a significant role for BSEP in the pathogenesis of NASH.

In a previous study using data from human biopsy samples, BSEP was downregulated during progression of NAFLD [27]. Therefore, we initially hypothesized that BSEP downregulation and subsequent bile acid accumulation in the liver contribute to NASH progression [27]. However, HFD-fed *Bsep*^*+/-*^ mice exhibited milder steatosis and less weight gain, compared with HFD-fed WT mice. Furthermore, the intrahepatic TG and cholesterol levels were reduced in HFD-fed *Bsep*^*+/-*^ mice, compared with HFD-fed WT mice.

Regarding bile acid metabolism, we initially hypothesized that *Bsep*^+/-^ mice have excess bile acid in the liver because BSEP facilitates bile acid excretion. However, the TBA level was reduced in the liver of HFD-fed *Bsep*^+/-^ mice, compared with the other mice. An HFD increases the fecal bile acid concentration [36, 37] and reduces the hepatic TBA concentration [38]. Indeed, the TBA level was reduced in HFD-fed mice, compared with ND-fed mice (Fig 2A). However, the mechanism by which TBA loss occurs in the liver of *Bsep*^+/-^ mice is unclear.

To explore the mechanism underlying TBA loss, we next analyzed the hepatic expression levels of genes related to bile acid metabolism. BSEP is a main export transporter of bile acid and is positively regulated by FXR, a receptor for bile acid. Heterozygous knockout of *Bsep* theoretically induces bile acid excess in the liver, leading to stimulation of FXR. FXR activates MRP2 and inactivates CYP7A1 and NTCP, thereby maintaining bile acid homeostasis in hepatocytes. The upregulation of *Fxr* and *Mrp2* expression levels in HFD-fed *Bsep*^*+/-*^ mice suggests a compensatory action against the downregulation of *Bsep*. In contrast, *Cyp7a1* (the rate-limiting enzyme in bile acid synthesis) and *Ntcp* (a bile acid uptake transporter), both of which contribute to intracellular TBA accumulation, were also upregulated in the liver of HFD-fed *Bsep*^*+/-*^ mice. The upregulation of *Cyp7a1* and *Ntcp* expression levels in HFD-fed *Bsep*^*+/-*^ mice was unexpected, but may be due to the loss of intracellular TBA caused by MRP2 upregulation, together with the HFD. Concerning bile acid fractions, the levels of the secondary bile acids TDCA, TLCA, and TUDCA were maintained in HFD-fed *Bsep*^*+/-*^ mice, irrespective of TBA reduction, supporting the hypothesis that bile acid uptake is increased by NTCP activation.

Concerning lipid metabolism, the hepatic cholesterol and TG concentrations were reduced in HFD-fed *Bsep*^+/-^ mice. We focused on the metabolism of cholesterol, a precursor of bile acids. *Hmgcr* (the rate-limiting enzyme in cholesterol synthesis), *Ldlr* (the main cholesterol uptake transporter), and *Srebf2* (a regulator of cholesterol metabolism) were upregulated in HFD-fed *Bsep*^+/-^ mice, compared with ND-fed *Bsep*^+/-^ mice. SREBP2 senses reductions in the cholesterol level in the liver and stimulates HMGCR and LDLR to maintain the cholesterol pool. These alterations appear to be reasonable compensative reactions, with respect to the reduced cholesterol level. Consequently, we speculate that the activation of cholesterol conversion to bile acid, which is mediated by the product of *Cyp7a1*, induces cholesterol loss in the liver; this results in the activation of *Hmgcr*, *Ldlr*, and *Srebf2*. The expression levels of *Abcg5*, the cholesterol export transporter, were similar among ND-fed *Bsep*^+/-^ mice, ND-fed WT mice, and HFD-fed WT mice; however, this level was significantly upregulated in HFD-fed *Bsep*^+/-^ mice. ABCG5 is regulated by two nuclear receptors: liver receptor homolog 1 (LRH1) [39] and hepatocyte nuclear factor 4 (HNF4a) [40]. Alterations in bile acid metabolism independent of SREBP2 may mediate *Abcg5* upregulation in the liver of HFD-fed *Bsep*^+/-^ mice. Furthermore, upregulation of ABCG5, the cholesterol export transporter, might reduce the intrahepatic lipid content in HFD-fed *Bsep*^+/-^ mice.

In addition, we analyzed the intestinal expression levels of genes related to bile acid and cholesterol metabolism. *Fxr* and its downstream gene *I-babp* were significantly upregulated in HFD-fed *Bsep*^*+/-*^ mice. We were unable to analyze the bile acid flow from the liver to the intestinal tract; however, our results suggested an increased concentration of bile acid in the intestinal tract, because FXR is a receptor for bile acid. Moreover, bile acid is a derivative of cholesterol; thus, activation of bile acid secretion from the liver might induce the conversion of cholesterol to bile acid.

Intestinal FXR is a key molecule for improvement of insulin sensitivity and liver metabolism [41, 42]. Furthermore, de Boer *et al*. reported that the activation of intestinal FXR induces transintestinal cholesterol excretion [43]. The *Fxr* upregulation observed in HFD-fed *Bsep*^*+/-*^ mice in the present study might improve systemic metabolism and induce cholesterol excretion through the intestine, thereby reducing both hepatic steatosis and weight gain. TGR5, another bile acid receptor, is highly expressed in several organs and has various roles in energy metabolism [18]. Among those roles, the activation of TGR5 in enteroendocrine cells leads to secretion of GLP‑1, resulting in improved pancreatic function, insulin secretion, and insulin sensitivity [44]. Intestinal *Tgr5* upregulation might also have improved glucose metabolism and induced a favorable phenotype in HFD-fed *Bsep*^*+/-*^ mice. Regarding intestinal cholesterol metabolism, *Npc1l1*, the cholesterol uptake transporter in the ileum, tended to be downregulated in HFD-fed *Bsep*^*+/-*^ mice. This result is consistent with the less severe steatosis observed in HFD-fed *Bsep*^*+/-*^ mice. BSEP is expressed in the liver [45], particularly on the bile canalicular membrane, but not in the intestine. The findings indicate that intrahepatic BSEP downregulation induces alterations in hepatic and intestinal bile acid and cholesterol metabolism, thereby suggesting that BSEP is closely associated with enterohepatic circulation and the gut–liver axis.

As discussed above, the downregulation of BSEP, as observed in our previous human study, did not result in progression of NAFLD in mice in the presence of an HFD alone, at least in terms of steatosis. However, focal inflammatory changes were observed both in ND- and HFD-fed *Bsep*^*+/-*^ mice, which is also a key factor of NAFLD progression. Similar changes were observed in a recently published paper by Fuchs *et al*. showing the attenuation of steatosis and aggravation of inflammation in the liver of BSEP-knockout mice with methionine choline-deficient (MCD) diet [46], although the observed inflammation is more evident compared with our present study. The difference of degree of inflammation may be attributed to the expression levels of BSEP (complete knockout or hetero knockout) or the difference of the diet. In any cases, BSEP down-regulation may contribute to NAFLD progression in terms of inflammation, not steatosis. In humans, the “multiple parallel hits” hypothesis states that various factors are associated with progression of NASH; these include lifestyle behaviors such as alcohol and exercise, diabetes mellitus, genetic factors, and dysbiosis [4]. Therefore, BSEP downregulation and HFD may be insufficient for progression of NAFLD; other factors were needed to induce NASH in the mice used in this study. Further investigations of those other factors and the underlying mechanism are therefore warranted.

In this study, BSEP downregulation may influence bile acid and upstream cholesterol metabolism in both the liver and intestine. Considering the changes in intestinal metabolism, fecal contents of bile acid, cholesterol, and the microbiome may be affected, which could contribute to the progression of NAFLD. Taken together, the findings of our previous study and the present study suggest an association of BSEP with NASH pathogenesis. Alternatively, *Bsep* downregulation induced favorable metabolic alterations in HFD-fed mice; these included improvement of steatosis and weight gain without causing major adverse reactions. Although the concept appears contradictory, BSEP may prevent the progression of steatosis in early stages of NAFLD, but not in NASH with inflammation. Further investigation of the roles of BSEP in the progression or amelioration of NAFLD could provide novel therapeutic options targeting bile acid metabolism, including transporters such as BSEP.

In conclusion, although progression of NAFLD was not observed, the downregulation of BSEP induced alterations in expression levels of genes associated with bile acid and lipid metabolism in both the liver and ileum in mice fed an HFD. Based on the “multiple parallel hits” hypothesis, additional unidentified factors are involved in the development of NASH in this mouse model; however, BSEP plays an important role in both NAFLD and NASH through modifications to bile acid and lipid metabolism. Further investigations of the functions of BSEP and other factors associated with progression of NAFLD will provide insight into NASH pathogenesis, resulting in the development of novel therapeutic strategies.

## Supporting information

S1 Fig Blood examinations. **A**: AST, **B**: ALT, **C**: ALP, **D**: T-CHO, **E**: TG, **F**: Glucose. Values are expressed as means ± standard deviations (n  = 4,  5, or 6). * *p* < 0.05.
(PDF)

S2 Fig Histological analyses of the liver related to inflammation and fibrosis. **A, B, C, and D**: Hematoxylin & eosin staining of representative liver specimens. **A and B**: ND-fed *Bsep*^*+/-*^ mice. C and D: HFD-fed *Bsep*^*+/-*^ mice. Bars: 200 μm (A and C) and 100 μm (B and D). **E, F, G, and H**: Azan staining of representative liver specimens. **D**: ND-fed WT mice. **E**: ND-fed *Bsep*^*+/-*^ mice. **F**: HFD-fed WT mice. **G**: HFD-fed *Bsep*^*+/-*^ mice. Bars: 500 μm (E, F, G, and H).
(PDF)

S3 Fig Hepatic expression levels of genes related to fibrosis, as determined by real-time PCR. **A, B, and C**: Hepatic expression levels of *Timp1*, *Mmp3*, and *Tgfb1*. Values are expressed as means ± standard deviations (n  =  5 or 6). * *p* < 0.05.
(PDF)

S4 Fig BSEP protein expression and localization, as determined by immunohistochemistry of the liver. **A, B, C, and D**: Immunohistochemistry with a BSEP antibody of representative liver specimens. A: ND-fed WT mice. B: ND-fed *Bsep*^*+/-*^ mice. C: HFD-fed WT mice. D: HFD-fed *Bsep*^*+/-*^ mice. Bars: 100 μm (A, B, C, and D).
(PDF)

S1 Table Primers and probes of reverse transcriptase-polymerase chain reaction. (XLSX)
