# Supplementary figures and images for: Heterozygous knockout of Bile salt export pump ameliorates liver steatosis in mice fed a high-fat diet
Source: PLoS One. 2020 Aug 12;15(8):e0234750. doi: 10.1371/journal.pone.0234750 (PMC7423142; doi:10.1371/journal.pone.0234750)

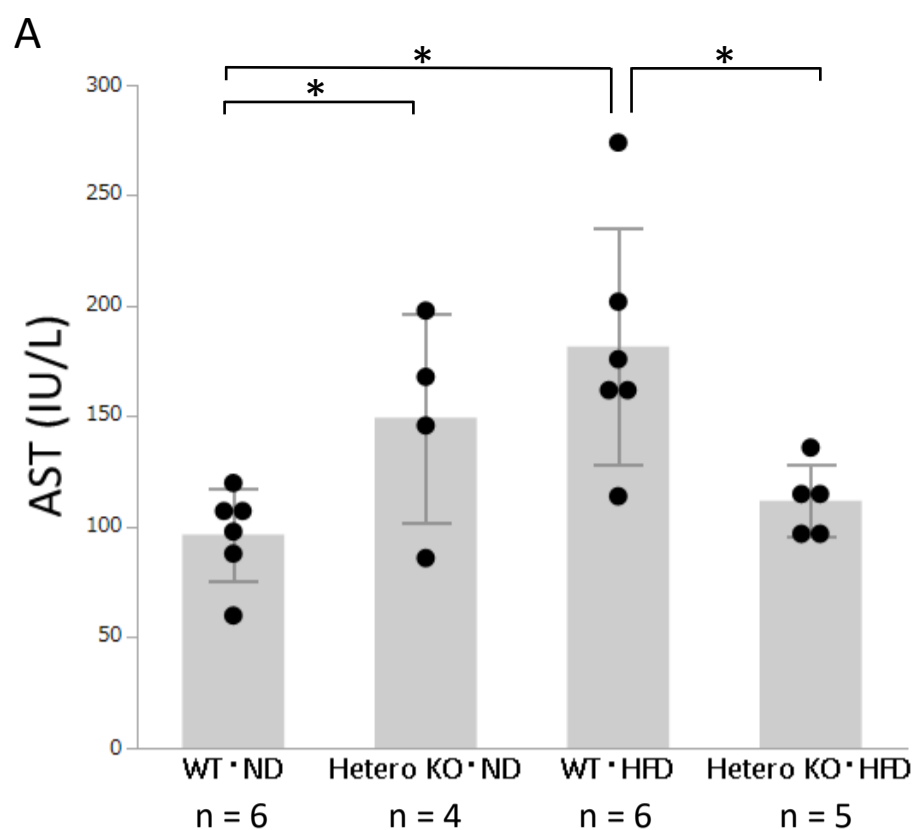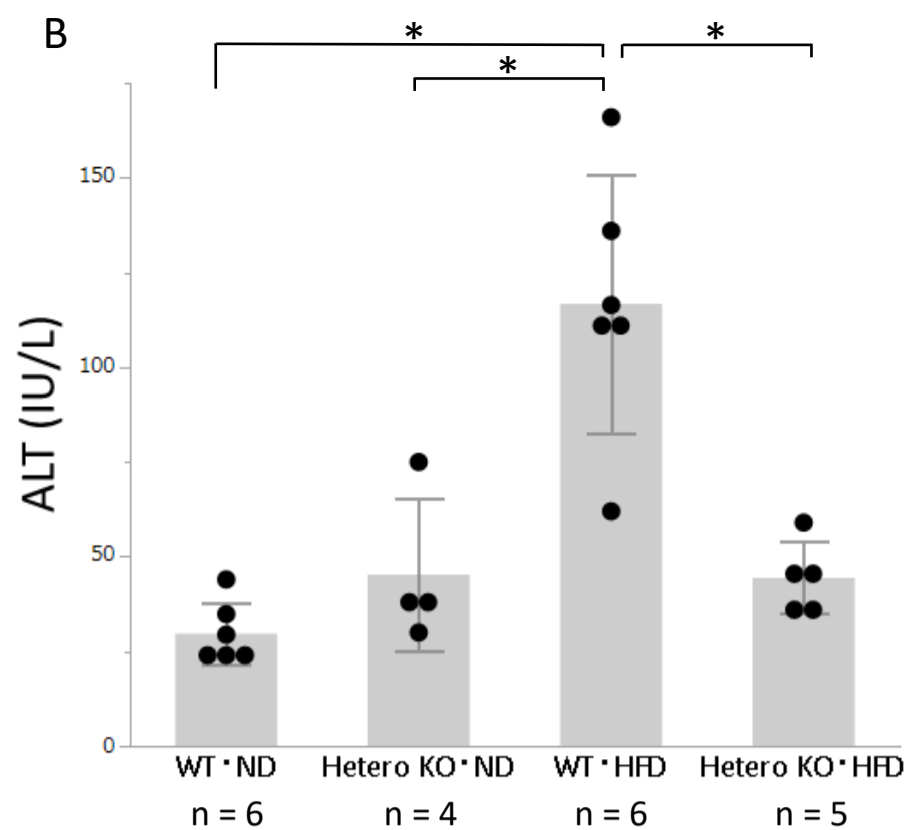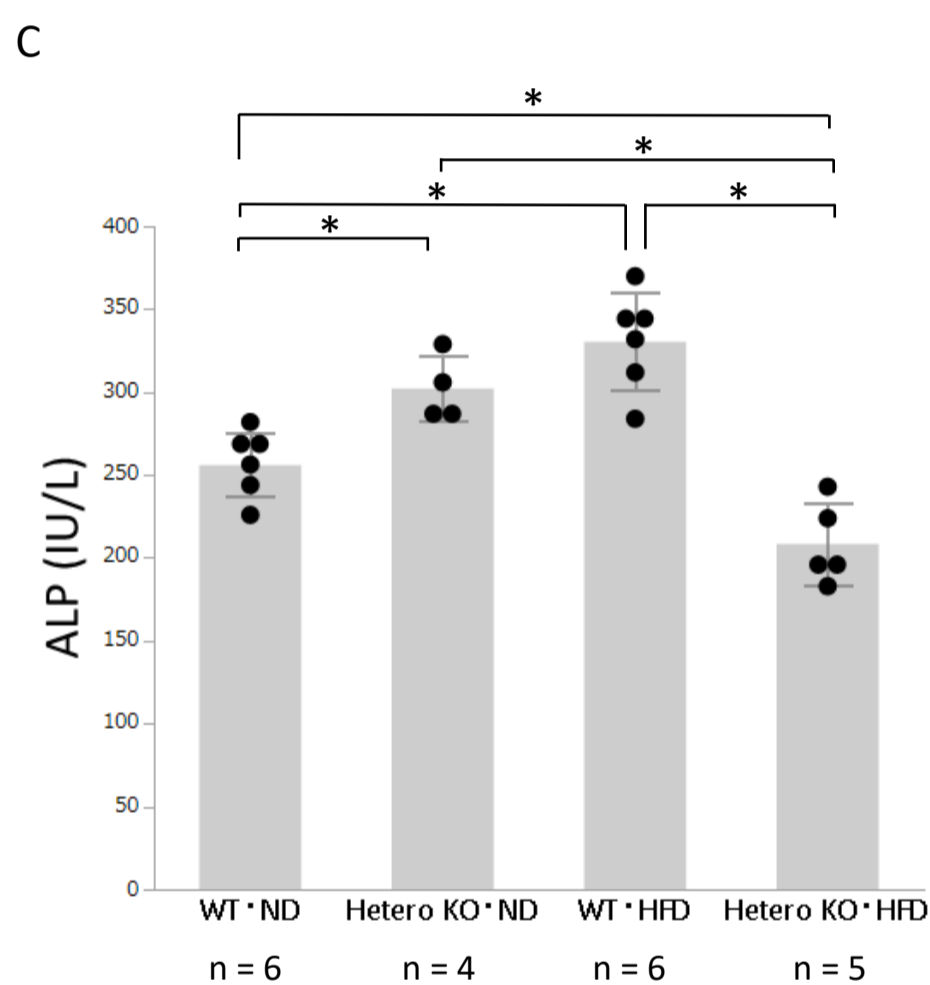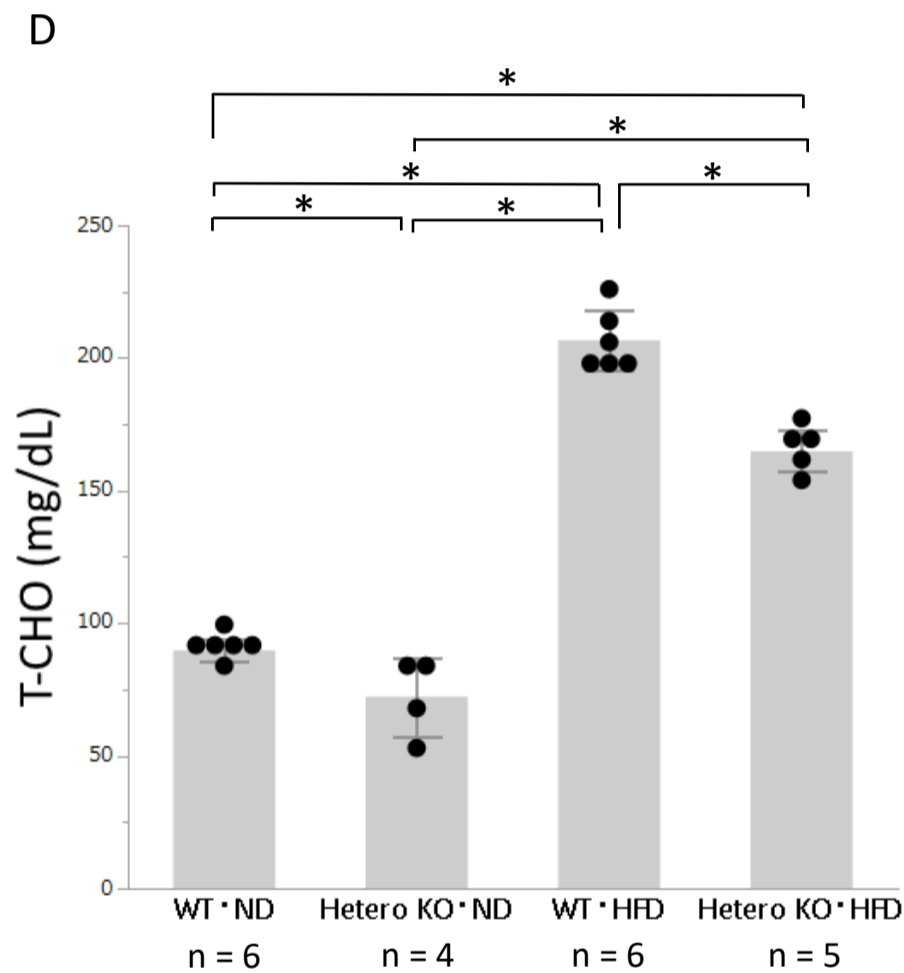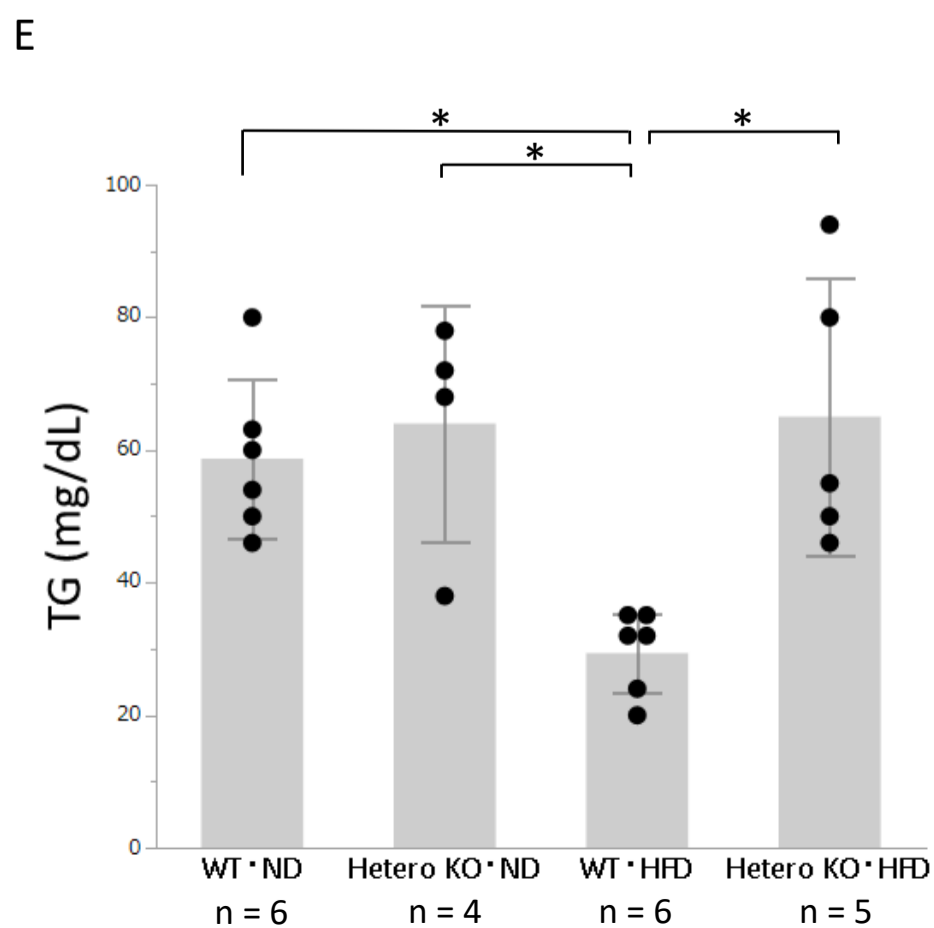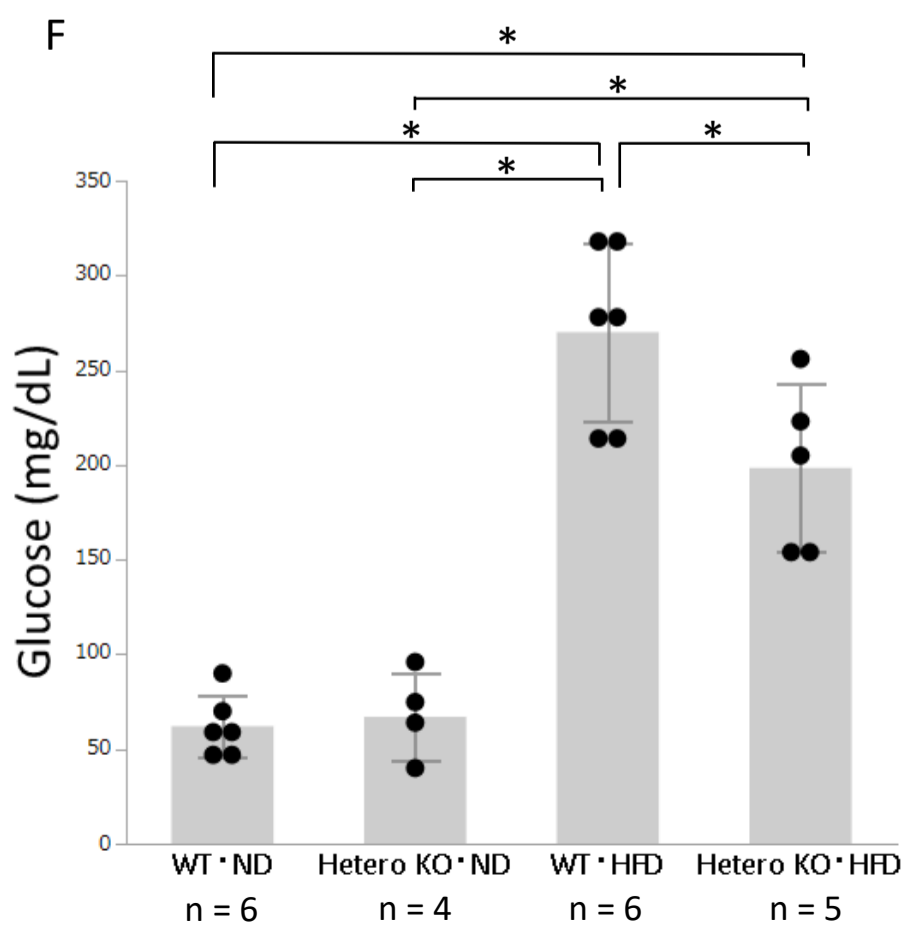

Supplement: S1 Fig — A: AST, B: ALT, C: ALP, D: T-CHO, E: TG, F: Glucose. Values are expressed as means ± standard deviations (n = 4, 5, or 6). * p < 0.05. (PDF) [file pone.0234750.s001.pdf]

A

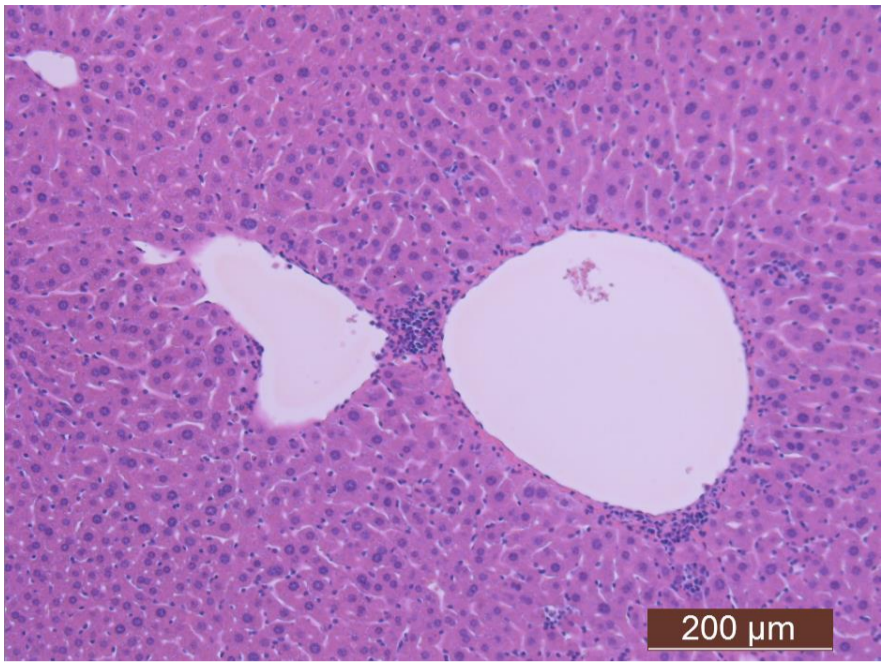

B

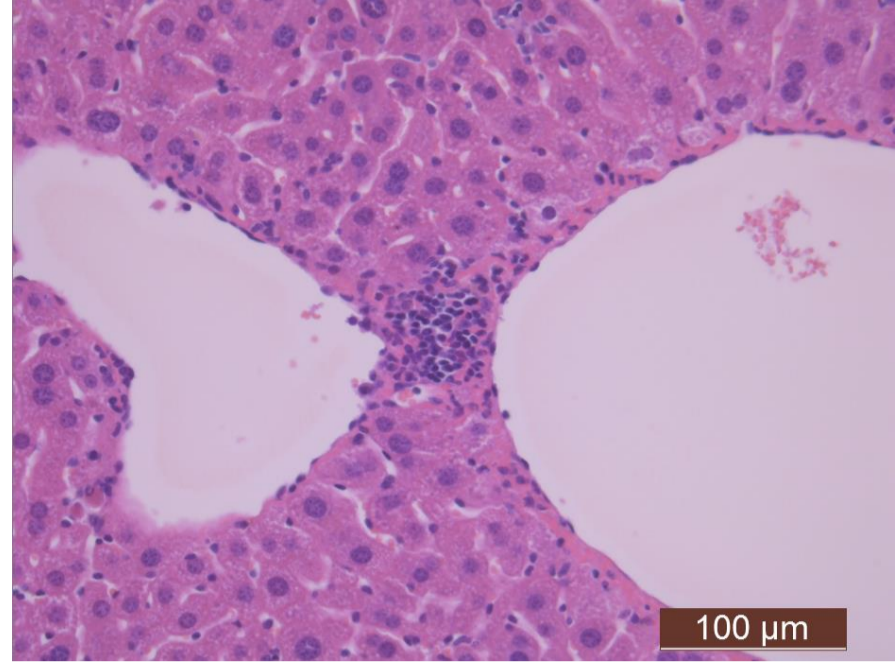

C

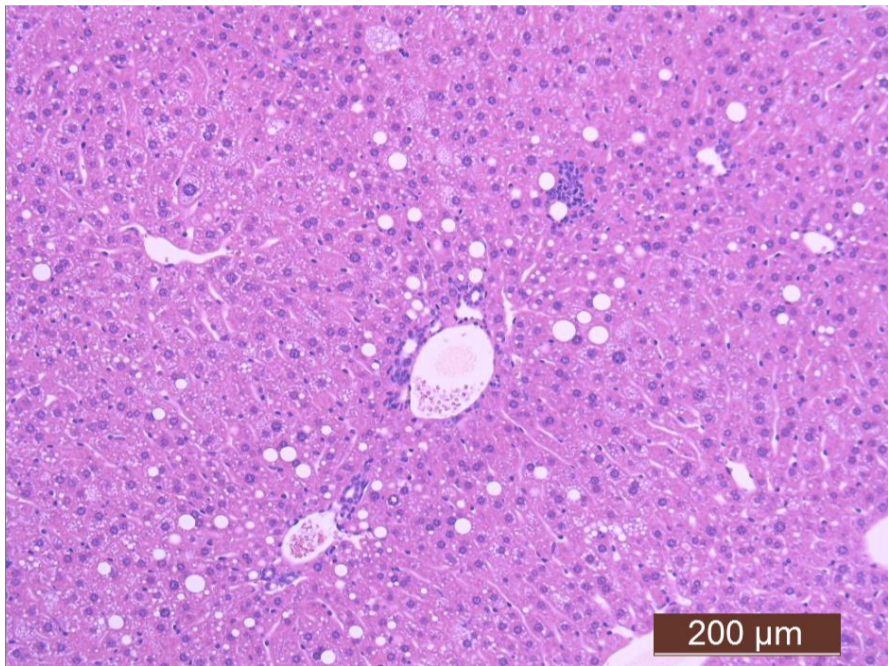

D

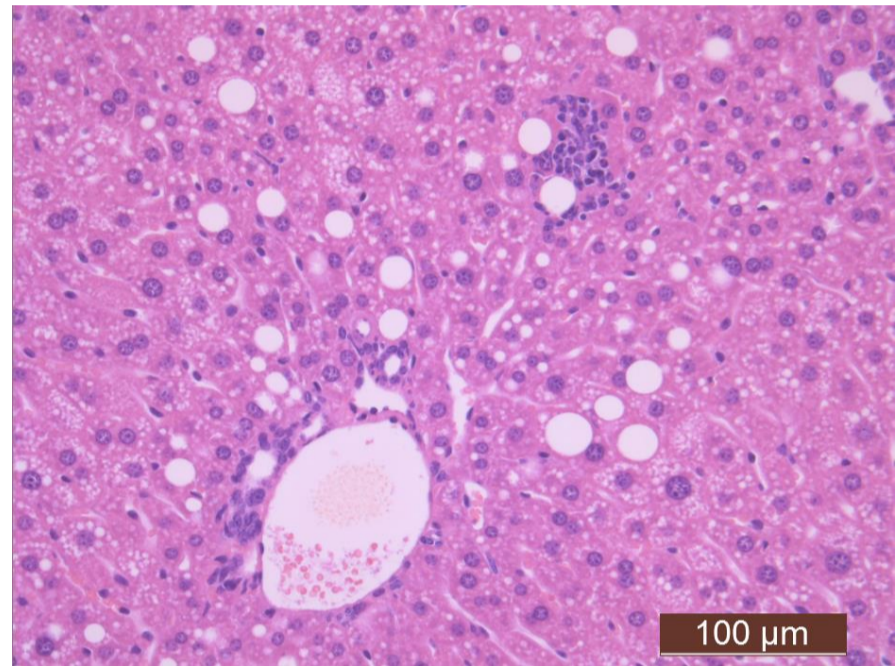

E

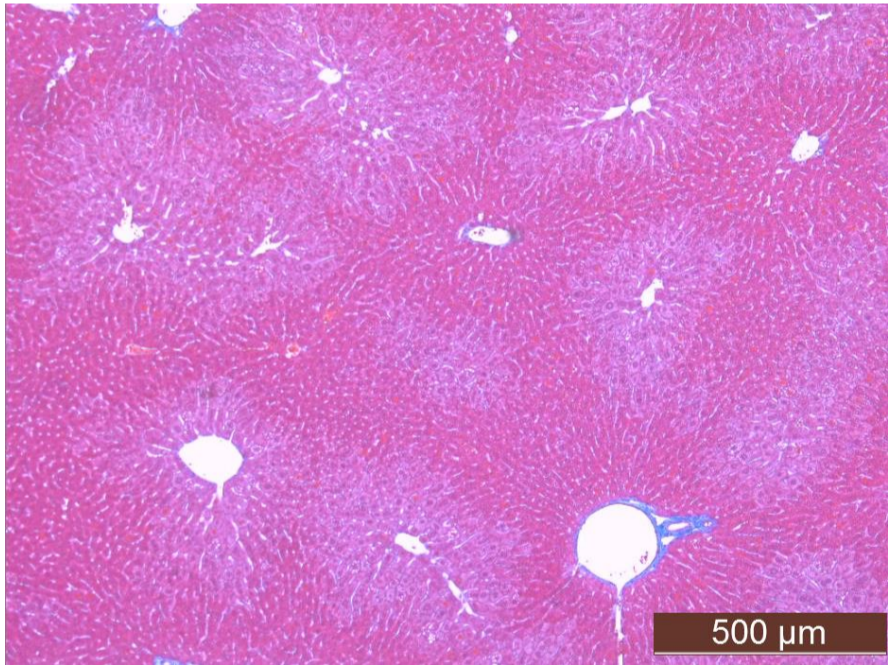

F

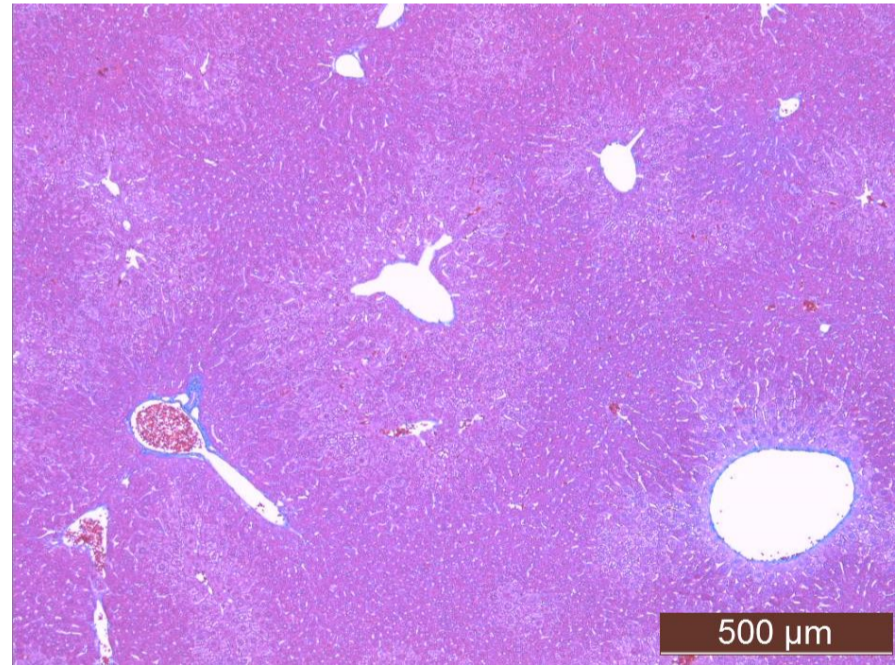

G

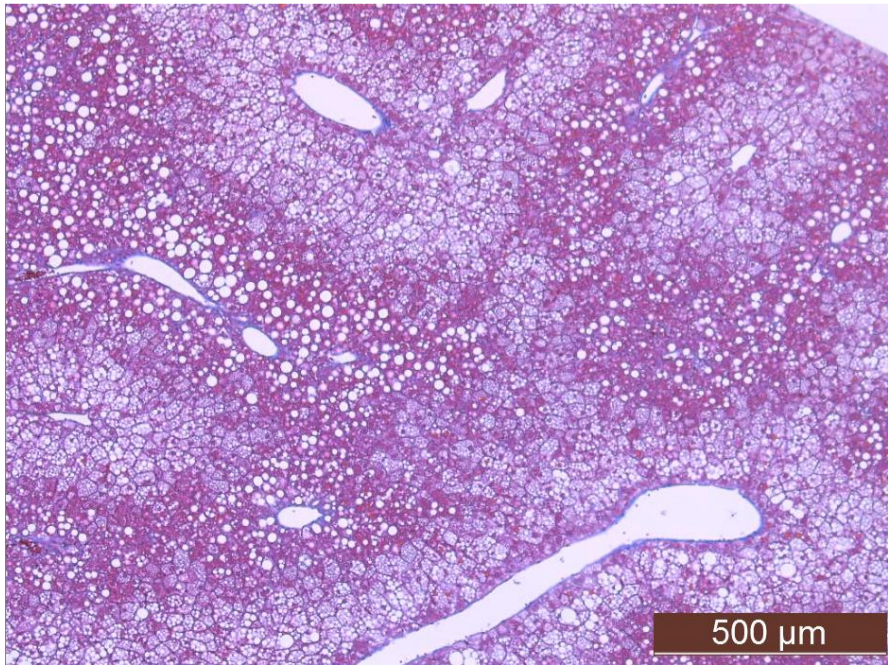

H

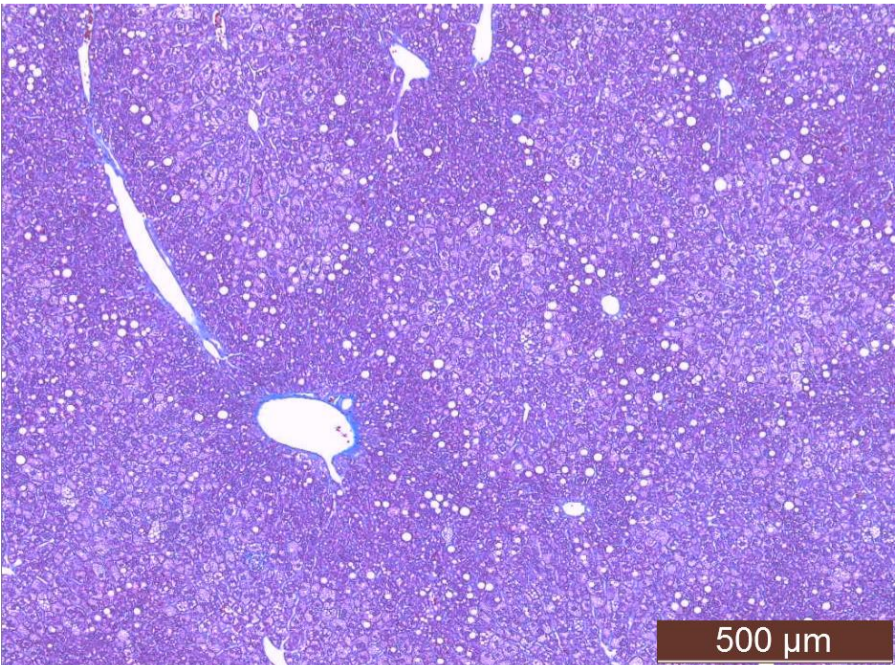

Supplement: S2 Fig — A, B, C, and D: Hematoxylin & eosin staining of representative liver specimens. A and B: ND-fed Bsep+/- mice. C and D: HFD-fed Bsep+/- mice. Bars: 200 μm (A and C) and 100 μm (B and D). E, F, G, and H: Azan staining of representative liver specimens. D: ND-fed WT mice. E: ND-fed Bsep+/- mice. F: HFD-fed WT mice. G: HFD-fed Bsep+/- mice. Bars: 500 μm (E, F, G, and H). (PDF) [file pone.0234750.s002.pdf]

A

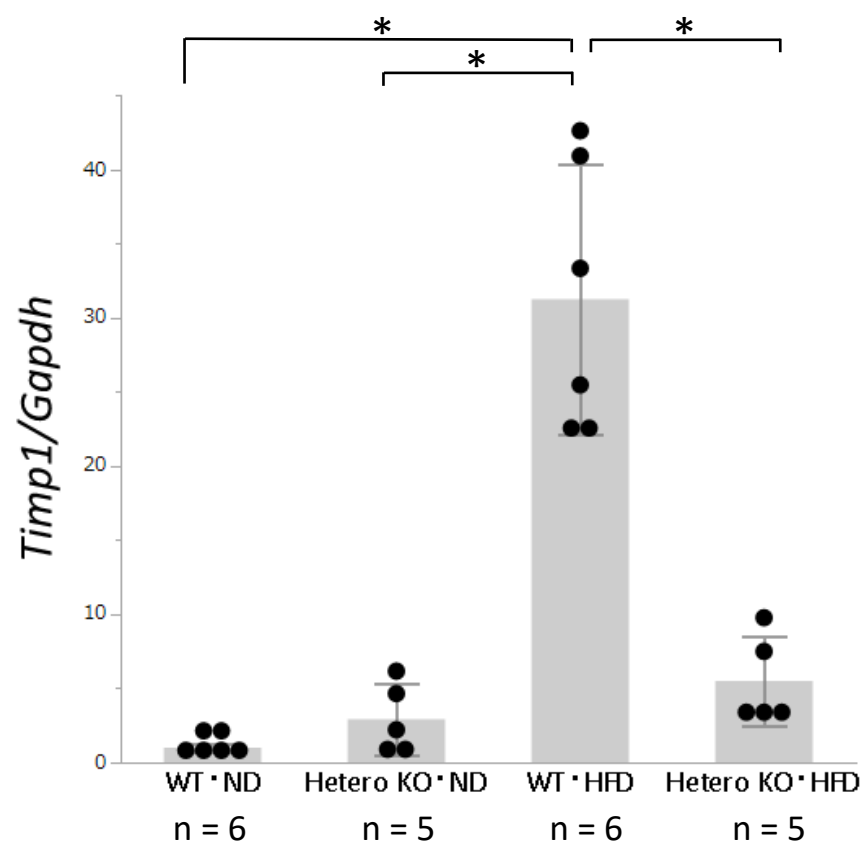

B

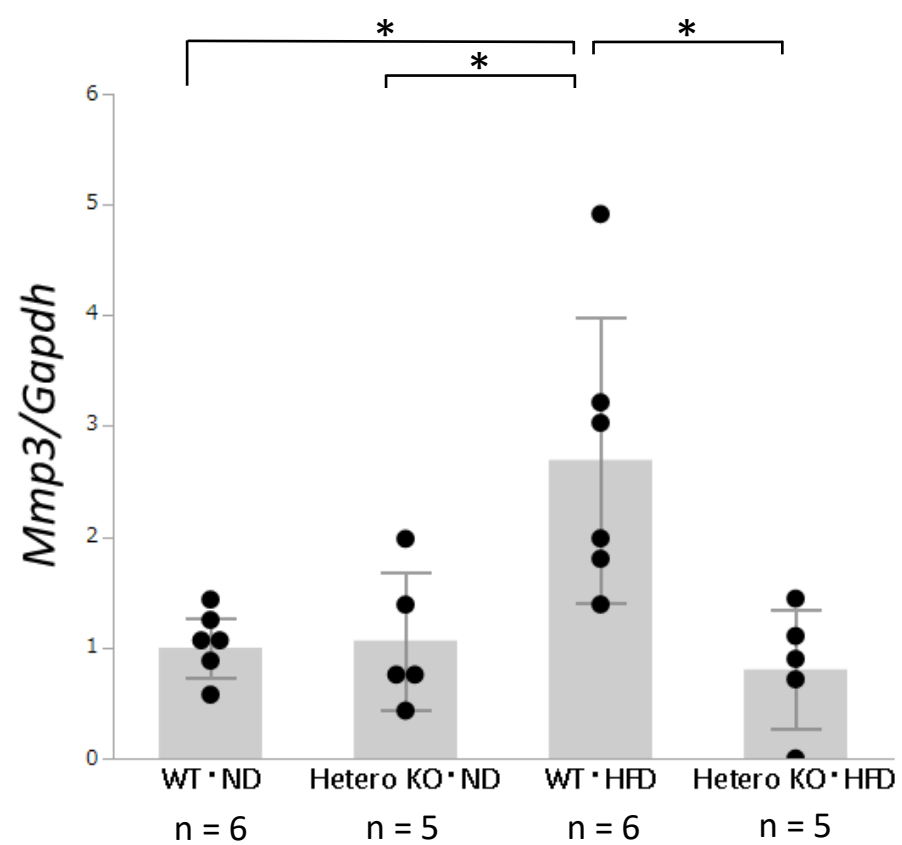

C

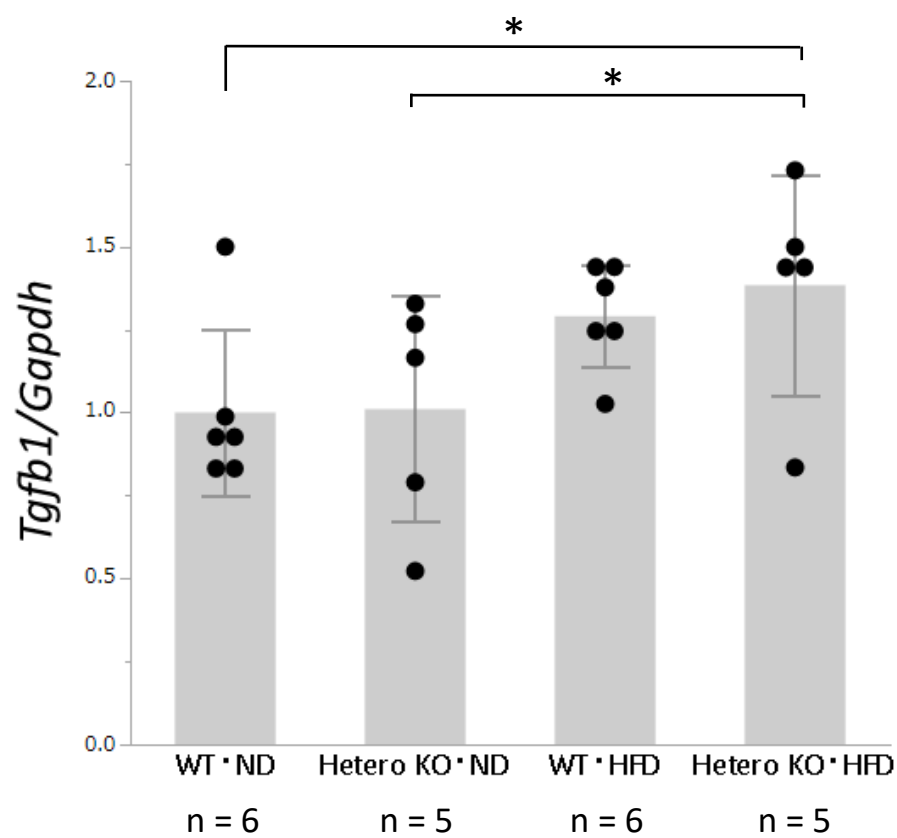

Supplement: S3 Fig — A, B, and C: Hepatic expression levels of Timp1, Mmp3, and Tgfb1. Values are expressed as means ± standard deviations (n = 5 or 6). * p < 0.05. (PDF) [file pone.0234750.s003.pdf]

A

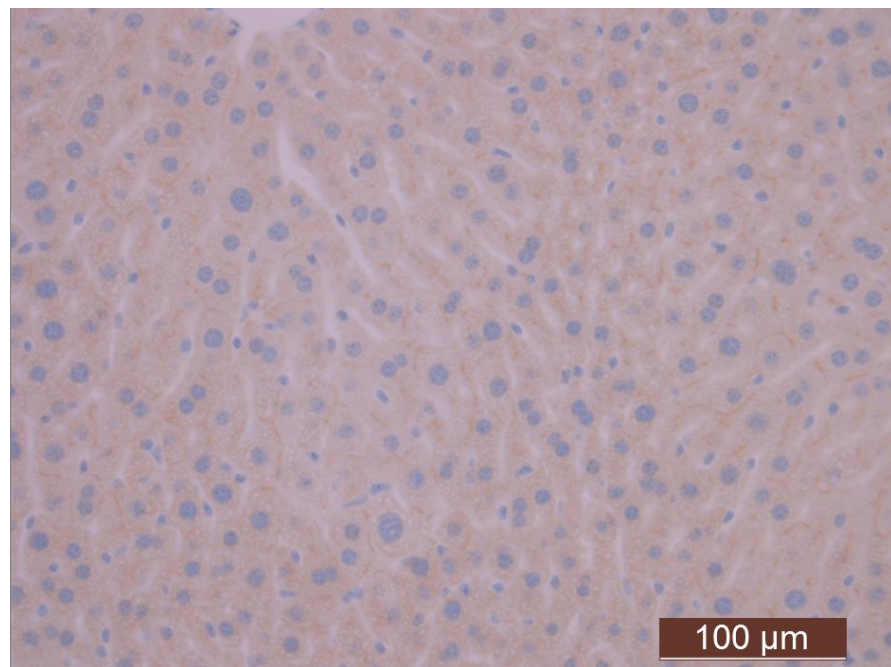

B

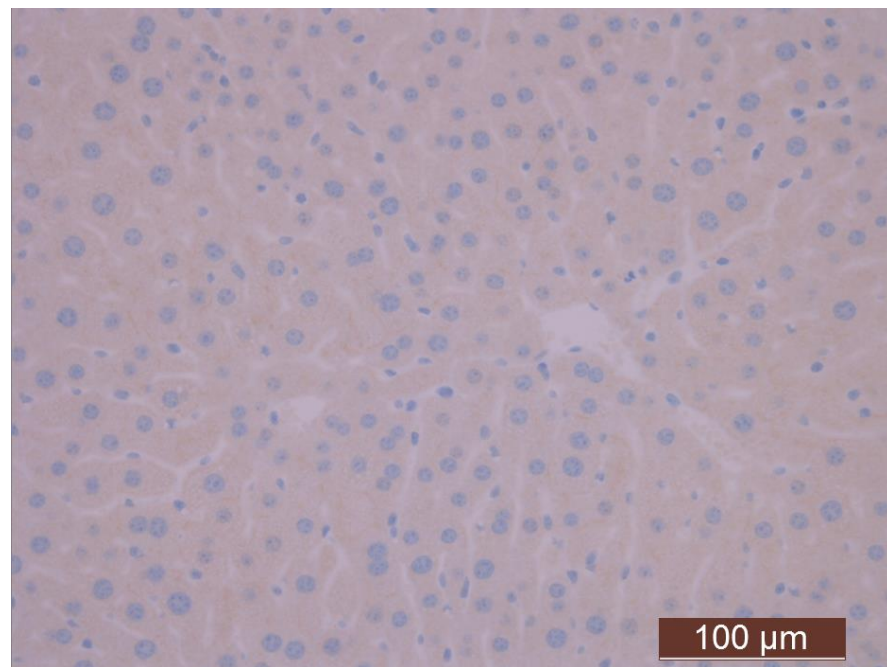

C

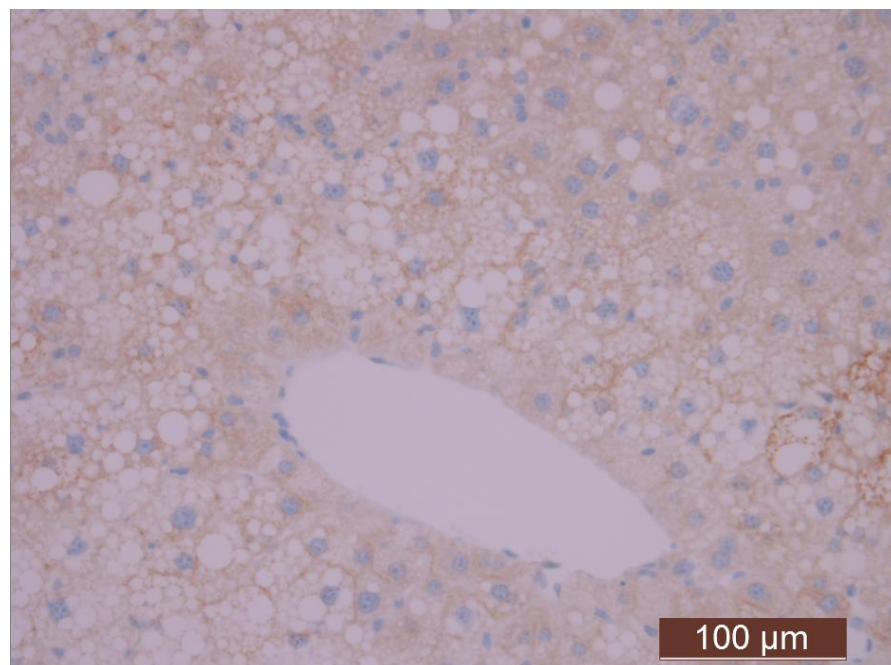

D

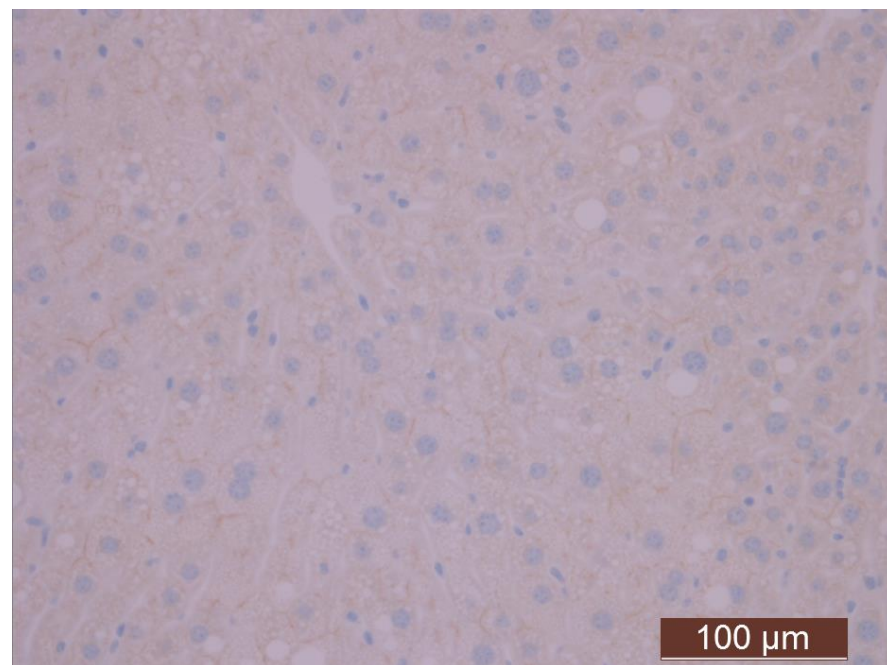

Supplement: S4 Fig — A, B, C, and D: Immunohistochemistry with a BSEP antibody of representative liver specimens. A: ND-fed WT mice. B: ND-fed Bsep+/- mice. C: HFD-fed WT mice. D: HFD-fed Bsep+/- mice. Bars: 100 μm (A, B, C, and D). (PDF) [file pone.0234750.s004.pdf]
